# A matter of nerves

**DOI:** 10.7554/eLife.75629

**Published:** 2021-12-23

**Authors:** Timothy J Duerr, James R Monaghan

**Affiliations:** 1 Department of Biology, Northeastern University Boston United States

**Keywords:** limb regeneration, size regulation, neural control, proportionality, axolotl, Other

## Abstract

Regrowing new body parts requires neural input to restore appropriately sized limbs in salamanders.

**Related research article** Wells KM, Kelley K, Baumel M, Vieira WA, McCusker CD. 2021. Neural control of growth and size in the axolotl limb regenerate. *eLife* **10**:e68584. doi: 10.7554/eLife.68584

The ability of humans to regrow lost or damaged body parts is extremely limited and pales in comparison to other animals, such as salamanders or planarians. For example, the axolotl salamander can regenerate full-sized body parts throughout its entire life (Stocum, 2017).

These animals grow new limbs through a structure known as the blastema, which forms at the end of the amputated limb and contains a diverse group of proliferating cells that contribute to the new limb (Gerber et al., 2018). The formation of the blastema and the initial response following amputation have been widely studied, but how the axolotl regenerates appropriately sized structures after an injury remains unclear. Now, in eLife, Catherine McCusker and colleagues at the University of Massachusetts Boston – including Kaylee Wells as first author – report new insights into the mechanisms that regulate limb size following amputation (Wells et al., 2021).

Wells et al. first identified different growth phases during limb regeneration after the blastema has formed. The ‘early tiny limb’ phase creates a small, new limb with the right structure and shape, and the ‘late tiny limb’ phase involves growing the limb until it has reached the right size. Wells et al. found that the two phases differed in the rate at which the limbs grew, with the early tiny limb phase regenerating tissue more rapidly than the later phase. Wells et al. show that the growth observed during these phases was due to a decrease in cell death and an increase in cell number and size.

Previous research has shown that severing the nerve supply to an amputated forelimb halts regeneration, indicating that an intact nerve supply is essential for limbs to regrow (Todd, 1823). Wells et al. thus explored the abundance of nerves in the early and late tiny limb phase. They found that the early tiny limb contained more nerve fibers, suggesting that nerves may also play a role in regulating the size of a regenerating limb. Indeed, cutting off the nerve supply to the limb during the early tiny limb phase reduced the production of new cells and increased the amount of dying cells.

Next, Wells et al. deviated a nerve from the shoulder onto an open wound made on either small or large animals to determine if the number of nerves dictates the size of the new limb. They then grafted differently sized blastemas from small or large animals onto the open wound. Wells et al. hypothesized that larger animals have more nerves, so even if a smaller blastema is grafted onto a large animal, the limb that regenerates would grow to the same size as the host’s existing limb. Indeed, large animals with smaller blastemas grew full-sized limbs, whereas small animals with large blastemas only grew smaller, size-matched limbs (Figure 1).

**Figure 1.**
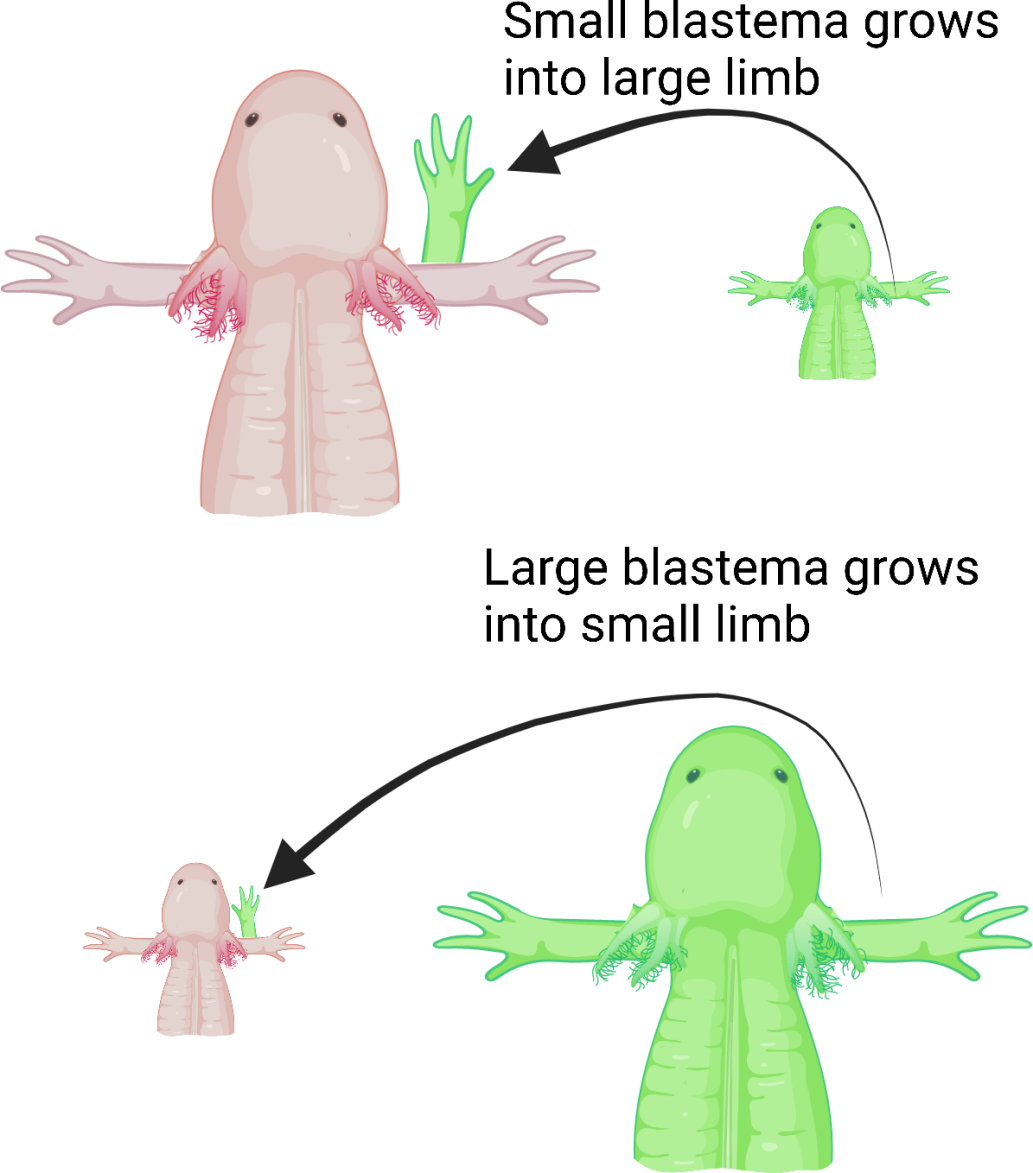
Limb regeneration in the axolotl salamander. Previous studies suggest that nerves play a role in limb regeneration. Wells et al. hypothesized that larger animals have more nerves, and that blastemas from a smaller animal grafted onto a large animal would cause the new limb to grow to the same size as the animal’s existing limb. Large (top; pink) and small (bottom; pink) axolotls were grafted with blastemas from small (top; green) and large (bottom; green) individuals, respectively. Regardless of the size of the blastema they received, the animals grew limbs matching their body size, suggesting that the number of nerves could play a role in determining the size of the regenerate limb.

Image credit: The figure was generated using BioRender.

Wells et al. then tried to determine whether the nerves or other components of the host’s limb environment are responsible for the size of the new body part. To do so, instead of deviating a nerve to the open wound of small and large animals, they grafted neuron bundles of the same size onto the injury site. Adding identically sized blastemas resulted in the animals growing a limb with matching stature, indicating that non-neuronal factors from the host have a limited impact on how big a leg regrows. However, Wells et al. also demonstrate that the number of nerves alone does not dictate the size of the regrown limb: animals of similar size and with similarly sized grafted blastemas also had identically sized limbs when they received neuron bundles with varying numbers of nerves.

Nerves are thought to be exclusively involved in motor control and somatosensory perception, but growing evidence shows they have other, more novel roles in the body, such as helping immune cells to develop and enabling wound healing and regeneration (Ordovas-Montanes et al., 2015; Pirotte et al., 2016). Wells et al. report evidence for another unique role for nerves in regulating the size of regrown limbs following amputation. The next obvious step is to identify the proteins supplied by the nerves that enable limbs to regenerate to an appropriate size (Singer, 1978). A better understanding of the proteins involved will be critical to determine if other animals, including humans, possess the ability to regenerate body parts, and how this could be exploited in regenerative medicine.
